# Administering selected subscales of patient-reported outcome questionnaires to reduce patient burden and increase relevance: a position statement on a modular approach

**DOI:** 10.1007/s11136-023-03587-8

**Published:** 2024-01-24

**Authors:** Daniel Serrano, David Cella, Don Husereau, Bellinda King-Kallimanis, Tito Mendoza, Tomas Salmonson, Arthur Stone, Alexandra Zaleta, Devender Dhanda, Andriy Moshyk, Fei Liu, Alan L. Shields, Fiona Taylor, Sasha Spite, James W. Shaw, Julia Braverman

**Affiliations:** 1 Pharmerit International, Bethesda, MD USA; 2 Northwestern University, Evanston, IL USA; 3 University of Ottawa, Ottawa, ON Canada; 4 LUNGevity Foundation, Bethesda, MD USA; 5 University of Texas MD Anderson Cancer Center, Houston, TX USA; 6 Consilium Salmonson & Hemmings, Uppsala, Sweden; 7 University of Southern California, Los Angeles, CA USA; 8 Independent Consultant, Philadelphia, PA USA; 9 Bristol Myers Squibb, Princeton, NJ USA; 10 Adelphi Values, Boston, MA USA; 11 Present Address: The Psychometrics Team, Sheridan, WY USA; 12 Present Address: Center for Cancer Research, National Cancer Institute at the National Institutes of Health, Bethesda, MD USA; 13 Present Address: CancerCare, New York, NY USA; 14 Present Address: Private Consultant, Escondido, CA USA; 15 Present Address: CSL Behring, King of Prussia, PA USA

**Keywords:** Modular approach, Patient-reported outcome, Oncology, Questionnaire, Clinical trial

## Abstract

Patient-reported outcome (PRO) questionnaires considered in this paper contain multiple subscales, although not all subscales are equally relevant for administration in all target patient populations. A group of measurement experts, developers, license holders, and other scientific-, regulatory-, payer-, and patient-focused stakeholders participated in a panel to discuss the benefits and challenges of a modular approach, defined here as administering a subset of subscales out of a multi-scaled PRO measure. This paper supports the position that it is acceptable, and sometimes preferable, to take a modular approach when administering PRO questionnaires, provided that certain conditions have been met and a rigorous selection process performed. Based on the experiences and perspectives of all stakeholders, using a modular approach can reduce patient burden and increase the relevancy of the items administered, and thereby improve measurement precision and eliminate wasted data without sacrificing the scientific validity and utility of the instrument. The panelists agreed that implementing a modular approach is not expected to have a meaningful impact on item responses, subscale scores, variability, reliability, validity, and effect size estimates; however, collecting additional evidence for the impact of context may be desirable. It is also important to recognize that adequate rationale and evidence (e.g., of fit-for-purpose status and relevance to patients) and a robust consensus process that includes patient perspectives are required to inform selection of subscales, as in any other measurement circumstance, is expected. We believe that the considerations discussed within (content validity, administration context, and psychometric factors) are relevant across multiple therapeutic areas.

## Plain English summary

The importance of patients’ perspectives in drug development, especially in cancer research, is increasing. This means that patients who participate in clinical trials are asked to fill out many questionnaires and surveys, which are used to measure aspects of the disease and treatment such as pain, fatigue, nausea, and functioning. However, some questions may be repetitive or not relevant to the patient’s condition, which can be burdensome and inefficient for the participants. To address this issue, a group of experts in measurement, drug development, patient advocacy, and scientific and regulatory matters came together to discuss ways to administer a subset of questionnaire items that would increase relevance and efficiency without compromising the validity of the results. This paper argues that, under certain conditions and with a rigorous selection process, it is acceptable and even preferable to use a subset of questions in patient-reported outcome measures in clinical trials.

## Introduction

Patient-reported outcome (PRO) questionnaires, whether developed using classical or modern methods or both, provide a unique opportunity to gather information on patients’ perspectives on the effect of treatment during clinical or registrational trials [1]. In this paper, we use the term *PRO measure* (PROM) to refer to a specific instrument completed as a self-report by a person with a specific clinical condition. For example, a person with non-small cell lung cancer completing the non-small cell lung cancer symptom assessment questionnaire (NSCLC-SAQ). In this example, the NSCLC-SAQ is the PROM. Throughout this paper, we use “PROM” singular and “PROMs” plural to refer to such instruments. PRO-based endpoints in oncologic research have increased over time with the recognition that survival is not always sufficient to characterize benefit/harm of oncologic treatment [2]. PROMs considered in this paper are multi-question inventories that assess multiple concepts (e.g., dyspnea, fatigue, etc.) across multiple subscales. Examples of such PROMs relevant in oncology include the European Organisation for Research and Treatment of Cancer Quality of Life Questionnaire Core 30 (EORTC QLQ-C30) and the Functional Assessment of Cancer Therapy-General (FACT-G). While only some subscales of such PROMs may be relevant to patients within a given context of use, it is routine in oncology trials to collect answers to all questions measuring all concepts regardless of relevance to patients. This approach can place unnecessary burdens on participants when questions are perceived to be redundant or not relevant to the patient condition [3]. Therefore, it is important to consider respondent burden in order to minimize missing data and, ultimately, to maximize the quality of patient-reported data to support treatment benefit decisions by regulators [4].

Administering the clinically relevant and patient-relevant subset of domains from a larger PROM within a specific context of use is described here as a “modular” approach, as further detailed below. We suggest that it is acceptable, and at times preferred, to take a modular approach when administering PROs in oncology clinical trials (and to other therapy areas, as applicable). For example, dyspnea would be a clinically relevant and patient-relevant domain to evaluate in lung cancer and much more proximal to the disease and treatment process than emotional functioning, thereby motivating a modular collection of the QLQ-C30 dyspnea domain over an exhaustive collection of the entire QLQ-C30. This position is based on the shared experiences and perspectives of stakeholders participating in a discussion panel that included experts in measurement and psychometrics, veterans of both health technology assessment (HTA) bodies and regulatory agencies in both Europe and the US, individuals with experience in patient advocacy and behavioral science, and developers/license holders of commonly used oncology-specific PRO questionnaires.

Within this position paper, we define our understanding of a modular approach and acknowledge the perceived barriers and risks in the oncology field and from other perspectives to applying a modular approach to PRO administration in interventional trials. We also evaluate whether a modular approach has impact on the content validity, psychometric performance, and interpretation of scores from the subscales selected for administration. We do not answer the question of how subscales are selected (i.e., identification of most important and relevant subscales to measure from the target patient population perspective), which is a fundamental consideration in the context of the goals of a specific study. Rather, we focus on methodological and statistical justification for using a modular approach. This paper should be perceived as an opinion paper aiming to encourage strategic thinking in the industry and facilitate the patient-centric drug development process. The paper is motivated by the prevalent practice of exhaustive PROM administration in randomized clinical trials when only a subset of PROM domains is relevant within a context of use. It is important to note that a modular approach does not necessarily mean the measurement of fewer concepts, but rather allowing for the flexibility in selecting optimal subscales across different instruments. We further note that while this paper is motivated by the need for specific guidelines on implementation of a modular approach, both COSMIN (https://www.cosmin.nl/research-publications/) and COMET [5] have provided guidance on the distinct but related questions of PRO evidence reporting and PRO core-set administration. While core sets are related to the modular approach discussed in this paper, they are distinct because core sets can be more broad, as detailed next.

## What is a modular approach?

**Fig. 1:**
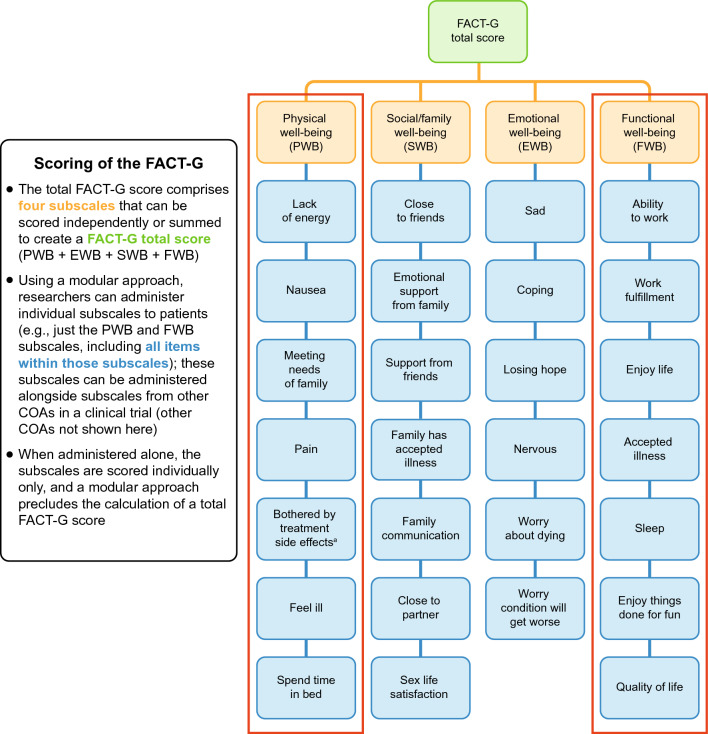
Administration of select subscales of the FACT-G using a modular approach. ^a^GP5 item within PWB subscale can be administered as a standalone item to assess the concept of perceived bother with side effects of treatment

We define a modular approach as collecting non-exhaustive but patient-relevant and clinically relevant domains from existing multi-domain PROMS within a given context of use that is independently scored, interpreted, and psychometrically validated for administration in each clinical trial. Modularization may require a subset of subscales from a given instrument or a mix of subscales from different measures. This definition is illustrated in Fig. 1 and, for example, displays an instance where researchers could administer all items from the physical well-being (PWB) and functional well-being (FWB) subscales of the FACT-G in an oncology trial, alongside subscales from other PROMs that are more relevant to the target patient population and treatment goals. An alternative example (not presented in the figure) would be to administer the physical functioning subscale from EORTC QLQ C30 along with the emotional and social well-being subscales from FACT-G. Please note that neither of these examples aim to make a suggestion for a specific PRO strategy, as the actual selection of instruments and subscales would depend on the specific study population, submission goals, and targeted reimbursement strategy.

Importantly, this definition of a modular approach does not imply the selection and administration of individual items within or across different subscales. Using the EQ-5D-5L as an example, administering only the “mobility” and “pain/discomfort” items is outside the scope of our definition of a modular approach, since all five EQ-5D-5L items (dimensions) are needed to calculate a health utility index. Nor do we define a modular approach as the use of an item bank to create a customized selection of items, or the selective analysis of specific subscales of a questionnaire administered in full, both of which have been described before [6]. Administering only certain items within a subscale raises challenges that are beyond the scope of this paper and requires that the single items and/or subsections of validated subscales/modules undergo psychometric evaluation. However, in cases where an individual item has been shown to be a valid measure of a concept and will be analyzed individually (rather than in combination with other items), it may be a relevant example of a modular approach as defined here. Using the GP5 item to measure the level of bother of treatment side effects is such an example, which is widely used in research and industry [7–9].

## The benefits and challenges with using a modular approach

A modular approach has potential advantages for patients enrolled in oncology trials as well as for researchers/sponsors. Even though there is evidence that PRO questionnaire length is not necessarily associated with low compliance [10], it was noted that brevity and relevance of questionnaires should be considered to reduce frustration and burden when questions are perceived to be redundant or not relevant to the patient condition [4, 11]. The importance of measuring PRO concepts that are relevant to the target population has been emphasized in the literature [4, 12]. There is also reduced administrative and record-retention burden at clinical sites, which helps to eliminate data waste (i.e., collecting data that are not informative for understanding specific treatment impact or patients’ well-being). It has been noted that PRO data from clinical trials are underreported and that a lot of collected PRO data are never published, which is considered unethical [13, 14]. A modular approach allows researchers and sponsors to create a fit-for-purpose PRO strategy that focuses on what is important to patients and assesses concepts that can be modified by the trial treatment. Such a targeted PRO strategy allows for streamlined development of evidence packages by sponsors for regulatory review, reimbursement submission, and clinicians who make treatment recommendations. More generally, there is also the potential for less missing data, leading to results that reflect the treatment impact more precisely and accurately. Kluetz et al. stated that *“The goal [of PRO measure selection] should be to achieve a comprehensive evaluation of the patient experience most affected by the therapy, while maximizing the relevance of individual questions and minimizing overall burden and duplication”* [15].

At the same time, PROM modularization may present challenges. From a patient advocacy perspective, without well-defined guidelines to determine subscale selection, there is a critical risk in excluding important patient outcomes. For example, there is a growing conversation around broadening the definition of tolerability in cancer clinical trials to better capture the patient experience, including its impact on work and social function [16]. Therefore, a robust understanding of what is important to patients is a fundamental consideration in any PRO selection process and in particular during implementation of a modular approach.

From the payer perspective, as the data collected during clinical and registrational trials are used in valuation decisions, a modular approach imposes limits on the comparability across new products. For example, if trial 1 includes one subscale of a multi-subscale COA, trial 2 includes a different subscale, and trial 3 includes both subscales, comparison of PRO data between all three trials by HTAs may become challenging. For PROs with a total score, selecting specific subscales would prevent the creation of a total score. Another concern, from the perspective of multiple stakeholders, is that a modular approach should only be considered where the definitions of clinically important difference (CID) and meaningful within-patient change (MWPC) are not affected (i.e., such information is available at the subscale level). These terms, related to Jaeschke, Singer, and Guyatt’s nomenclature for “minimal important difference” or “change” [17, 18], defining the ability of a PROM to reflect true change arising organically or from treatment efficacy, have been defined in the US Food and Drug Administration (FDA) guidance [19, 20] and European Medicines Agency [21] reflection papers.

Finally, from the developer and licensing perspective, administering selected subscales is appropriate only when they have been psychometrically evaluated for administration (for a specific population and context of use). In some cases, this evidence may be generated in parallel with a trial. A related point is that using select subscales out of a questionnaire often requires permission from the individual license holders/developers.

## Content validity of assessment

Content validity is defined as the extent to which an assessment comprehensively measures concepts that are relevant to a disease and important to patients with the condition, in ways that the respondent can understand and to which they can provide a meaningful response [22–24]. Many measures were developed with the specific intention to capture multiple domains of experience because, for example, the desire was to measure health-related quality of life as operationalized by multiple subscales. In other cases, measures may have been developed to capture all possible facets of symptom burden that has been validated for a broader population than the one that will undergo evaluation. For example, while the EORTC QLQ-C30 subscales have shown evidence of validity and relevance for the general oncology population [25], some of the subscales may not be equally relevant and valid for specific patients and new treatments. Pain, for example, is the prominent symptom for pancreatic cancer, but is much less relevant for patients with lymphoma who are not living with other pain-related comorbidities. Moreover, as new treatments emerge, previously reported side effects may lose their relevance. For example, vomiting and insomnia are typical side effects for chemotherapy, but not for recently emerged chimeric antigen receptor T-cell treatments. Thus, a modular approach may be required to maintain content validity as the context of use evolves or is changed.

Content validity is of concern to both regulators and HTA bodies in demonstrating the efficacy, safety, and cost-effectiveness of new therapies. An evaluation that prioritizes content across all subscales contained in a measure so that only those subscales deemed relevant to the investigational agent, and comparator can guide the selection process. If the criteria for individual subscale content validity are met, then, given guidance [22], we would expect regulators and HTA bodies to accept a modular approach. In fact, the FDA encourages the administration of more targeted measures [26], and the European Medicines Agency also emphasizes the need to determine the relative importance of different PROM domains a priori [27]. Evidence demonstrating the satisfaction of these criteria needs to be provided to all stakeholders explaining the selection of modules/subscales, with particular attention given to the different assessment needs of regulators versus payers.

From a payer perspective, thorough assessment of symptoms and adverse events is important for comparisons between treatments in different studies (noting that comparability is already somewhat limited because not all data are reported or made publicly available). A move to a modular approach would, therefore, require some additional guidance to the payers and other stakeholders. However, and as summarized in Brogan et al., two key factors that influence payer decisions in terms of acceptability of PRO data are the extents to which (1) relevant results were generated from well-controlled clinical studies and presented transparently, and (2) the PROM itself has been psychometrically evaluated in the target patient population and published in the peer-reviewed literature [28]. Neither of these factors would preclude the modular approach discussed herein as far as the evidence of validation that is available for the HTA review.

From a patient and patient advocacy perspective, it is fundamental that domains which are highly relevant to the patient experience are captured within trials. Patients want to share what they believe to be the most relevant concepts related to their condition, and they want this information to be used in treatment decisions and drug development and valuation. Furthermore, patients are generally willing to answer as many questions as needed when they receive and support the reasoning for the inclusion of questions [3]. To support the inclusion of relevant domains of patient experience that foster content validity, steps must be taken to enhance efficiency and reduce redundancy in PRO administration, which may contribute to decreased motivation and item completion, particularly among individuals with low health literacy level or cognitive impairment [4, 29, 30]. For example, the EORTC QLQ-C30 instrument and PRO-CTCAE items are frequently administered together in oncology clinical trials, but contain items that assess similar concepts such as pain, depression/anxiety, and nausea. The traditional PRO administration approach obliges patients to answer multiple questions on each of these concepts. A modular approach may help to reduce overlap if there are no redundant items in the selected subscales. As another example, if the Patient-Reported Outcomes Measurement Information System (PROMIS) Cognitive Function short scale is administered to assess cognitive impairment, it may not be necessary to administer cognitive function items from EORTC QLQ-C30 if both instruments are used in the same trial.

Of note, content validity is inherently tied to the measurement domain selection process. The domain selection process is beyond the scope of this paper, but it is important to consider that content validity is central in that decision-making and tied to the goals of a specific study, and should involve engagement with all key stakeholders [26–28]. If a modular approach to PROM selection is taken, there is the potential for biased selection of concepts that may mask negative impacts of therapy or unintentionally omit outcomes that can inform valuation. Of note, concerns over biased selection of concepts are not unique to a modular approach and can be addressed in part by selecting subscales a priori and justifying their selection. In this way, the selection of subscales for administration is no different from selecting content valid PRO questionnaires to support prespecified endpoints in a clinical trial. Furthermore, the selection of individual subscales is analogous to selecting individual items from PRO-CTCAE, a common and FDA-recommended practice, and therefore, similar guidance should be used to avoid bias in the selection of subscales [31, 32].

## Contextual importance of subscale administration

The order of items on the questionnaire creates a context, or meaning, for the entire questionnaire. There is evidence that items placed early in the questionnaire affect the way in which people respond to later questions [33]. Therefore, an important consideration is whether responses to items on a subscale—for example, one that falls toward the end of a questionnaire—will vary if completed as a standalone subscale compared with the subscale embedded in the entire questionnaire. Although there is evidence to suggest that contextual variables may matter in assessment, the magnitude of those effects tends to be small, and may vary according to the questionnaire administration features and content. For example, in a study that evaluated placing a self-rated general health question before and after chronic health items (e.g., diagnosis of asthma or heart disease) in a survey, the presence of an ordering effect differed by language. Specifically, the only significant effect of question order was seen the Spanish group [34]. No order effect was observed when investigating three commonly used PROMs in an oncology setting, or in the specific case of assessing head and neck cancer using the FACT system [35, 36].

Due to conflicting examples of instances when context did and did not make a difference to response patterns, along with there being no regulatory guidance on the order of questionnaires when multiple measures are used within a clinical trial, empirical evidence may be useful to garner support for a modular approach. However, evidence to support contextual equivalence may only be needed in the short term, like how the field is now more accepting of mode-of-administration equivalence [37].

## Psychometric performance and score interpretation

Psychometric performance is broadly defined here as the demonstrated behavior of scores produced by an assessment when administered in the target patient population. Generally, instruments are developed and validated using traditional methods under which characteristics of the subscales (e.g., internal consistency and known-groups validity) are estimated and reported independently for each subscale. This contrasts with estimation of these quantities within a multidimensional item response theory (MIRT) framework, in which subscale interdependencies can be accounted for [38]. Resulting from the use of traditional methods, modular administration of, for example, the QLQ-C30 fatigue domain, will not systematically alter the psychometric properties.

As discussed in the previous sections, for content-valid subscales, if we assume that the order of administration is unlikely to impact patient responses, then subscale score averages, variability, reliability, validity, ability to detect change, and CID and MWPC definitions would not be expected to change appreciably or meaningfully in a target patient population (compared with administration of the entire questionnaires). In fact, it is possible that these estimates may improve due to decreased burden of assessment to the respondent, although evidence may be needed to confirm this. If selecting subscales from within an instrument, the scoring of individual subscales and score interpretation guidelines will not change (assuming that the subscales are scored independently); only total scores would be impacted (if applicable), and we do not recommend creating total scores if only select modules are present. In addition, certain psychometric properties, like conditional independence, can eliminate order effects.

It is important to note that order effects and the concerns related to them are largely eliminated within the modern psychometric framework. This is derived from the item conditional independence resulting from the estimated item parameters that condition the items on the latent variable, thereby orthogonalizing the items. Note that conditional independence of items only holds true to the extent that local dependence among items has been evaluated during calibration, and any detected dependence resolved [39]. For example, under fully calibrated item banks, like PROMIS or computer adaptive tests, custom instruments can be administered from the bank without regard to order of item administration [40, 41].

From an HTA perspective, payers may have concerns over the impact a modular approach has on the ability to calculate total scores for a PROM. However, the use of individual subscale scores is common in oncology; for example, the total score for the EORTC QLQ-C30 was introduced more than a decade after the EORTC QLQ-C30 became available, and has been used only rarely since then [42]. Both CID and MWPC definitions need to be available at the subscale level when using a modular approach. For example, if CIDs are defined for individual subscales (e.g., physical or role functioning [EORTC QLQ-C30]; physical, emotional, functional, or social well-being [FACT-G]; and urinary symptoms [EORTC prostate (QLQ-PR25)]), administration of those subscales in the absence of the remaining subscales is unlikely to affect the interpretation of the subscales.

From the developer/licensing perspective, the notion that a PROM can be “validated” is anathema to the exercise, considering that validation refers to cumulative evidence and is typically germane to a given context or patient population as opposed to a binary issue (i.e., whether a PROM has or has not been validated). Validation is a conclusion in context, so the additional validation evidence always adds value irrespective of whether a modular approach is implemented. In other words, in terms of psychometric validity, the modular approach would not be different from any other PRO strategy and selection of PROMs in general. Multi-scaled PRO-based endpoints are often based on subscale-level scores rather than total summary scores, with each subscale analyzed independently [43]. Because of limitations arising from methodological constraints at the time of development (e.g., EORTC), or aiming for efficiency in developing unidimensional measures (e.g., PROMIS), the frequently used PROMs perhaps unintentionally opened the door to the administration of specific subscale(s) by not directly estimating interdomain association. Therefore, as different domains of questionnaires are not mutually dependent, the premise that the entire questionnaire must be administered for the psychometric properties to be maintained is specious.

From a regulatory perspective, and consistent with the view expressed above, the Core Patient-Reported Outcomes in Cancer Clinical Trials Guidance for Industry: Draft Guidance (2021) from the FDA encourages the administration of relevant subscales to lessen patient burden: “*In some cases, subscales or subsets of questions from existing PRO instruments may be used to inform the benefit/risk assessment and support labeling claims if prospectively defined and their measurement properties have been adequately evaluated,*” adding that, *“When using a modular approach where these elements are able to be assessed and analyzed separately, different assessment frequencies can be selected that can reduce the response burden to patients”* [26]. Investigators can, of course, administer all subscales but only analyze a subset of the data, but there is questionable value in administering subscales that are not planned for analysis.

## Future use of modular approach

Despite being discussed for over a decade [44], the modular approach has not yet been widely implemented in interventional trials. An early example of a modular approach implemented in oncology clinical trials was the adaptation of the EORTC breast cancer–specific module (QLQ-BR23), in consultation with the EORTC, for use in four neoadjuvant and adjuvant studies [45]. In this example, the modification involved the removal of the arm and breast symptom scales (only relevant in the metastatic setting), which is akin to selecting the remaining functional and systemic therapy side effects scales for administration using a modular approach. The barriers against the more widespread implementation of this approach have been discussed above, including concern about contextual impact, performance validity, subscale selection bias, and impact on comparability across different trials.

Future research that gathers empirical evidence to influence broad support of the use of a modular approach is recommended until this methodology becomes well established in the field. While not discussed in this position paper, the selection of items for administration within a subscale is a future topic of interest to explore, which comes with its own set of considerations, especially ensuring that patients are being asked to share what is most relevant to their experience. Finally, although many of the topics presented here are transferable to therapeutic areas outside of oncology, consideration for application of a modular approach to PROM administration in other therapeutic areas should be further explored.

## Conclusion

The use of a modular approach to PROM administration is acceptable and does not compromise the validity of the selected subscales, with the following conditions:

1. Evidence is provided (including a well-defined process that includes patient engagement) on why subscales were selected.
2. Contextual impacts associated with subscale ordering have been considered/evaluated.
3. The PRO is scored at the subscale level, and subscale scores have been analyzed and reported in the literature for existing studies.
4. Subscale scores have been validated, including the availability of subscale-specific CID and MWPC definitions.
5. Permission of the developer/license holder has been obtained.

These recommendations are consistent with guidance from the FDA and the expressed willingness to accept this from a European regulatory perspective. Acceptance of a modular approach by the HTA community remains to be seen. This will likely require further education and evidence to show the comparability of a modular versus a traditional approach.

## References

[1] Mercieca-Bebber R, King MT, Calvert MJ, Stockler MR, Friedlander M (2018). The importance of patient-reported outcomes in clinical trials and strategies for future optimization. Patient Related Outcome Measures.

[2] Coleman RL, Beck JT, Baranda JC, Jacobs I, Smoyer KE, Lee LJ, Askerova Z, McGinnis J, Ganti AK (2021). The use of patient-reported outcome measures in phase I oncology clinical trials. Oncology.

[3] Atkinson TM, Schwartz CE, Goldstein L, Garcia I, Storfer DF, Li Y, Zhang J, Bochner BH, Rapkin BD (2019). Perceptions of response burden associated with completion of patient-reported outcome assessments in oncology. Value in Health.

[4] Aiyegbusi OL, Roydhouse J, Rivera SC, Kamudoni P, Schache P, Wilson R, Stephens R, Calvert M (2022). Key considerations to reduce or address respondent burden in patient-reported outcome (PRO) data collection. Nature Communications.

[5] Williamson PR, Altman DG, Bagley H, Barnes KL, Blazeby JM, Brookes ST, Clarke M, Gargon E, Gorst S, Harman N, Kirkham JJ, McNair A, Prinsen CAC, Schmitt J, Terwee CB, Young B (2017). The COMET handbook: version 1.0. Trials.

[6] Regnault A, Pompilus F, Ciesluk A, Mazerolle F, Bejar R, Fram RJ, Faller DV, Marquis P, Bell JA (2021). Measuring patient-reported physical functioning and fatigue in myelodysplastic syndromes using a modular approach based on EORTC QLQ-C30. Journal of Patient-Reported Outcomes.

[7] Pearman TP, Beaumont JL, Mroczek D, O'Connor M, Cella D (2018). Validity and usefulness of a single-item measure of patient-reported bother from side effects of cancer therapy. Cancer.

[8] Griffiths P, Peipert JD, Leith A, Rider A, Morgan L, Cella D, Cocks K (2022). Validity of a single-item indicator of treatment side effect bother in a diverse sample of cancer patients. Supportive Care in Cancer.

[9] US Food and Drug Administration & Critical Path Institute. Session 4: From individual symptoms to overall side effect burden. Presented at: Second Annual Workshop on Clinical Outcome Assessments in Cancer Clinical Trials; April 25, 2017; Bethesda, MD, USA

[10] Rolstad S, Adler J, Ryden A (2011). Response burden and questionnaire length: Is shorter better? A review and meta-analysis. Value in Health.

[11] Ettridge K, Caruso J, Roder D, Prichard I, Scharling-Gamba K, Wright K, Miller C (2021). A randomised online experimental study to compare responses to brief and extended surveys of health-related quality of life and psychosocial outcomes among women with breast cancer. Quality of Life Research.

[12] Retzer A, Calvert M, Ahmed K, Keeley T, Armes J, Brown JM, Calman L, Gavin A, Glaser AW, Greenfield DM, Lanceley A, Taylor RM, Velikova G, Brundage M, Efficace F, Mercieca-Bebber R, King MT, Kyte D (2021). International perspectives on suboptimal patient-reported outcome trial design and reporting in cancer clinical trials: A qualitative study. Cancer Medicine.

[13] Cruz Rivera S, Mercieca-Bebber R, Aiyegbusi OL, Scott J, Hunn A, Fernandez C, Ives J, Ells C, Price G, Draper H, Calvert MJ (2021). The need for ethical guidance for the use of patient-reported outcomes in research and clinical practice. Nature Medicine.

[14] Kyte D, Retzer A, Ahmed K, Keeley T, Armes J, Brown JM, Calman L, Gavin A, Glaser AW, Greenfield DM, Lanceley A, Taylor RM, Velikova G, Brundage M, Efficace F, Mercieca-Bebber R, King MT, Turner G, Calvert M (2019). Systematic evaluation of patient-reported outcome protocol content and reporting in cancer trials. Journal of the National Cancer Institute.

[15] Kluetz PG, Slagle A, Papadopoulos EJ, Johnson LL, Donoghue M, Kwitkowski VE, Chen WH, Sridhara R, Farrell AT, Keegan P, Kim G, Pazdur R (2016). Focusing on core patient-reported outcomes in cancer clinical trials: Symptomatic adverse events, physical function, and disease-related symptoms. Clinical Cancer Research.

[16] Basch, E., Campbell, A., Hudgens, S., Jones, L., King-Kallimanis, B., Kluetz, P., O'Connor, D., & Rosen, O. Broadening the definition of tolerability in cancer clinical trials to better measure the patient experience. A Friends of Cancer Research white paper. https://www.focr.org/sites/default/files/Comparative%20Tolerability%20Whitepaper_FINAL.pdf.

[17] Jaeschke R, Singer J, Guyatt GH (1989). Measurement of health status. Ascertaining the minimal clinically important difference. Controlled Clinical Trials..

[18] Schünemann HJ, Guyatt GH (2005). Commentary—Goodbye M(C)ID! Hello MID, Where do you come from?. Health Services Research.

[19] US Food and Drug Administration. Methods to identify what is important to patients & select, develop or modify fit-for-purpose clinical outcomes assessments. https://www.fda.gov/media/116277/download.

[20] US Food and Drug Administration. Patient-focused drug development: Incorporating clinical outcome assessments into endpoints for regulatory decision-making. https://www.fda.gov/media/166830/download.

[21] European Medicines Agency. Reflection paper on the regulatory guidance for the use of health related quality of life (HRQL) measures in the evaluation of medicinal products https://www.ema.europa.eu/en/documents/scientific-guideline/reflection-paper-regulatory-guidance-use-health-related-quality-life-hrql-measures-evaluation_en.pdf.

[22] US Department of Health and Human Services, US Food and Drug Administration, Center for Drug Evaluation and Research, Center for Biologics Evaluation and Research, & Center for Devices and Radiological Health. *Guidance for industry patient-reported outcome measures: Use in medical product development to support labeling claims.* Silver Spring, MD, USA: 2009.

[23] Shields AL, Hao Y, Krohe M, Yaworsky A, Mazar I, Foley C, Mehmed F, Globe D (2016). Patient-reported outcomes in oncology drug labeling in the United States: a framework for navigating early challenges. American Health and Drug Benefits.

[24] Terwee CB, Prinsen CAC, Chiarotto A, Westerman MJ, Patrick DL, Alonso J, Bouter LM, de Vet HCW, Mokkink LB (2018). COSMIN methodology for evaluating the content validity of patient-reported outcome measures: A Delphi study. Quality of Life Research.

[25] Cocks K, Wells JR, Johnson C, Schmidt H, Koller M, Oerlemans S, Velikova G, Pinto M, Tomaszewski KA, Aaronson NK, Exall E, Finbow C, Fitzsimmons D, Grant L, Groenvold M, Tolley C, Wheelwright S, Bottomley A (2023). Content validity of the EORTC quality of life questionnaire QLQ-C30 for use in cancer. European Journal of Cancer.

[26] US Department of Health and Human Services, US Food and Drug Administration, Oncology Center of Excellence, Center for Drug Evaluation and Research, & Center for Biologics Evaluation and Research. Core patient-reported outcomes in cancer clinical trials: Guidance for industry – draft guidance. Retrieved from https://www.fda.gov/regulatory-information/search-fda-guidance-documents/core-patient-reported-outcomes-cancer-clinical-trials. Retrieved February 4, 2022.

[27] European Medicines Agency. Appendix 2 to the guideline on the evaluation of anticancer medicinal products in man: The use of patient-reported outcome (PRO) measures in oncology studies. EMA/CHMP/292464/2014. http://www.ema.europa.eu/docs/en_GB/document_library/Other/2016/04/WC500205159.pdf.

[28] Brogan AP, DeMuro C, Barrett AM, D'Alessio D, Bal V, Hogue SL (2017). Payer perspectives on patient-reported outcomes in health care decision making: Oncology examples. Journal of Managed Care Specialty Pharmacy.

[29] Roa W, Brasher PMA, Bauman G, Anthes M, Bruera E, Chan A, Fisher B, Fulton D, Gulavita S, Hao C, Husain S, Murtha A, Petruk K, Stewart D, Tai P, Urtasun R, Cairncross JG, Forsyth P (2004). Abbreviated course of radiation therapy in older patients with glioblastoma multiforme: A prospective randomized clinical trial. Journal of Clinical Oncology.

[30] Calvert MJ, Cruz Rivera S, Retzer A, Hughes SE, Campbell L, Molony-Oates B, Aiyegbusi OL, Stover AM, Wilson R, McMullan C, Anderson NE, Turner GM, Davies EH, Verdi R, Velikova G, Kamudoni P, Muslim S, Gheorghe A, O'Connor D (2022). Patient reported outcome assessment must be inclusive and equitable. Nature Medicine.

[31] Kluetz, P. G., Chingos, D. T., Basch, E. M., & Mitchell, S. A. (2016). Patient-reported outcomes in cancer clinical trials: Measuring symptomatic adverse events with the National Cancer Institute’s patient-reported outcomes version of the Common Terminology Criteria for Adverse Events (PRO-CTCAE). *American Society of Clinical Oncology Educational Book,**35*, 67–73. 10.1200/edbk_159514 10.1200/EDBK_159514 27249687

[32] Smith AW, Mitchell SA, Aguiar CK, Moy C, Riley WT, Wagster MV, Werner EM (2016). News from the NIH: Person-centered outcomes measurement: NIH-supported measurement systems to evaluate self-assessed health, functional performance, and symptomatic toxicity. Translational Behavioral Medicine.

[33] Strack, F. “Order effects” in survey research: activation and information functions of preceding questions. In: Schwarz N, Sudman S, eds. *Context effects in social and psychological research* New York, NY, USA: Springer New York; 1992:23–34.

[34] Lee S, Grant D (2009). The effect of question order on self-rated general health status in a multilingual survey context. American Journal of Epidemiology.

[35] Novotny PJ, Dueck AC, Satele D, Frost MH, Beebe TJ, Yost KJ, Lee MK, Eton DT, Yount S, Cella D, Mendoza TR, Cleeland CS, Blinder V, Basch E, Sloan JA (2022). Effects of patient-reported outcome assessment order. Clinical Trials.

[36] Yount S, List M, Du H, Yost K, Bode R, Brockstein B, Argiris A, Vokes E, Cohen E, Campbell B, Valenzuela V, George J, Egan R, Chen J, Meddis D, Cella D (2007). A randomized validation study comparing embedded versus extracted FACT Head and Neck Symptom Index scores. Quality of Life Research.

[37] Muehlhausen W, Byrom B, Skerritt B, McCarthy M, McDowell B, Sohn J (2018). Standards for instrument migration when implementing paper patient-reported outcome instruments electronically: Recommendations from a qualitative synthesis of cognitive interview and usability studies. Value in Health.

[38] McDonald, R. P. *Test theory: a unified treatment.* Psychology Press; 2013.

[39] Chen W-H, Thissen D (1997). Local dependence indexes for item pairs using item response theory. Journal of Educational and Behavioral Statistics.

[40] Varni JW, Thissen D, Stucky BD, Liu Y, Gorder H, Irwin DE, DeWitt EM, Lai JS, Amtmann D, DeWalt DA (2012). PROMIS Parent Proxy Report Scales: An item response theory analysis of the parent proxy report item banks. Quality of Life Research.

[41] Reeve BB, Hays RD, Bjorner JB, Cook KF, Crane PK, Teresi JA, Thissen D, Revicki DA, Weiss DJ, Hambleton RK, Liu H, Gershon R, Reise SP, Lai JS, Cella D (2007). Psychometric evaluation and calibration of health-related quality of life item banks: Plans for the Patient-Reported Outcomes Measurement Information System (PROMIS). Medical Care.

[42] Giesinger, J. M., Kieffer, J. M., Fayers, P. M., Groenvold, M., Petersen, M. A., Scott, N. W., Sprangers, M. A. G., Velikova, G., Aaronson, N. K., & EORTC Quality of Life Group (2016). Replication and validation of higher order models demonstrated that a summary score for the EORTC QLQ-C30 is robust. Journal of Clinical Epidemiology.

[43] Gnanasakthy A, Barrett A, Evans E, D'Alessio D, Romano CD (2019). A review of patient-reported outcomes labeling for oncology drugs approved by the FDA and the EMA (2012–2016). Value in Health.

[44] Rothman M, Burke L, Erickson P, Leidy NK, Patrick DL, Petrie CD (2009). Use of existing patient-reported outcome (PRO) instruments and their modification: the ISPOR Good Research Practices for Evaluating and Documenting Content Validity for the Use of Existing Instruments and Their Modification PRO Task Force report. Value in Health.

[45] US Food and Drug Administration, & Critical Path Institute. Workshop on clinical outcome assessments (COAs) in cancer clinical trials. Retrieved from https://c-path.org/wp-content/uploads/2016/06/2016_coa_session2slides.pptx. Retrieved on June 15, 2021.

